# Adaptive profiles of Nellore sheep with reference to farming system and season: physiological, hemato-biochemical, hormonal, oxidative-enzymatic and reproductive standpoint

**DOI:** 10.1016/j.heliyon.2021.e07117

**Published:** 2021-05-26

**Authors:** D. Karthik, J. Suresh, Y. Ravindra Reddy, G.R.K. Sharma, J.V. Ramana, G. Gangaraju, P. Pandu Ranga Reddy, Y. Pradeep Kumar Reddy, D. Yasaswini, M.J. Adegbeye, P. Ravi Kanth Reddy

**Affiliations:** a Department of Livestock Production Management, College of Veterinary Science, Sri Venkateswara Veterinary University, Tirupati, India; b Center for Continuing Veterinary Education & Communication, College of Veterinary Science, Sri Venkateswara Veterinary University, Tirupati, India; c Department of Veterinary and Animal Husbandry Extension Education, College of Veterinary Science, Sri Venkateswara Veterinary University, Tirupati, India; d Controller of Examination, College of Veterinary Science, Sri Venkateswara Veterinary University, Tirupati, India; e Livestock Research Station, Sri Venkateswara Veterinary University, Palamaner, India; f Department of Animal Genetics and Breeding, College of Veterinary Science, Sri Venkateswara Veterinary University, Proddatur, India; g Department of Veterinary Medicine, College of Veterinary Science, Sri Venkateswara Veterinary University, Tirupati, India; h Department of Animal Science and Livestock Production, College of Agriculture and Natural Sciences, Joseph Ayo Babalola University, Ikeji-Arakeji, P.M.B 5006, Ilesha, Osun State, Nigeria; i Veterinary Assistant Surgeon, Animal Husbandry Department, Veterinary Dispensary, Taticherla, Prakasam District, Andhra Pradesh, India

**Keywords:** Adaptive profile, Farming system, Season, Sheep, Physiological parameters, Hematological parameters, Biochemical parameters, Oxidative enzymatic activity, Reproductive parameters

## Abstract

This paper outlines the effect of farming systems with reference to season on the body condition score (BCS) and adaptive profile (physiological, hemato-biochemical, hormonal, enzymatic and reproductive parameters) of Nellore sheep. In trial 1, sixty ewe-lambs were allotted to extensive, semi-intensive, and intensive rearing systems (n = 20) and evaluated for BCS at puberty, mating, 2 weeks pre-lambing and 2, 4, 8, and 12 weeks post-lambing. In trial 2, eighteen rams were distributed evenly to three farming systems (n = 6) and evaluated for physiological, hemato-biochemical, hormonal, enzymatic, and reproductive parameters concerning three seasons. Although the scores did not differ among the groups, the Kruskal-Wallis ranks of BCS revealed a higher energy status of intensive ewes at different physiological conditions. The sheep reared under extensive and semi-intensive systems displayed higher temperature, pulse rate and respiratory rate with predominant effects in summer season. Similarly, both systems exhibited higher WBC and lower haemoglobin, PCV, and RBC contents without affecting MVC, MCH, MCHC, and differential leucocyte count. The percent haemoglobin and RBC count were higher in winter compared to summer months, whereas WBC count followed an exactly opposite pattern. The sheep reared in intensive system showed higher glucose, total protein, albumin, cholesterol, T3, T4, calcium, and phosphorus; however, the globulin, creatinine, uric acid, aspartate amino transferase (AST), alanine amino transferase (ALT), superoxide dismutase (SOD), malondialdehyde (MDA), glutathione peroxidase, and catalase levels were elevated in extensive and semi-intensive systems. The dartos muscle extension (DME) and scrotum sweating rate (SSR) were higher for the sheep reared under extensive system, especially during summer season; while the seminal parameters viz., total sperm count, progressive sperm motility, and plasma membrane integrity were lower for extensive and semi-intensive sheep. No interactions were noticed for any of the parameters, except for cortisol, DME, and SSR, which showed significant interactions for rearing system vs. season. Our results showed dynamic adaptive mechanisms of the Nellore sheep in relation to different stressors like grazing for long distances, inadequate nutrition, and heat stress, revealing the heat resilient ability in harsh environmental conditions. Further, the analyzed vector plot showed that the AST, GPx, Cortisol, SOD, Catalase, WBC, PR, T4, total abnormalities, and major abnormalities were the major contributors for adapting during combined stressors.

## 1 Introduction

Livestock rearing plays a vital role in the economy of developing countries. Small ruminant farming provides income and employment to the poor households of rural society, especially in areas with hills, cliffs, and sparse vegetation. Sheep utilizes grass, browse, and agro-industrial byproducts more efficiently and valorizes the waste and biomass to energy and value-added products (Adegbeye et al., 2020). The prevailing food shortage and malnutrition problems of third world countries could be lessened by encouraging the sheep rearing practices.

The farming systems of sheep are broadly categorized into extensive, semi-intensive, and intensive rearing systems. Intensive system involves stall-feeding without sending the sheep for grazing; however, the semi-intensive and extensive systems allow the sheep to graze in an open environment for long hours. Grazing for long periods under high solar loads may trigger heat stress, and managing animals under low plane of nutrition induce nutritional stress. The impact of multiple stressors on grazing sheep should be considered, as it is unlikely that sheep will be exposed to a single stressor (Lees et al., 2019). The body weight gains, growth rates, and cost-effectiveness of the sheep under three farming systems are presented in Karthik et al. (2021). The same paper dealt with the heat and nutritional stress instigated by extensive farming system, leading to the lower body weight gains and lesser returns to the shepherds.

Stressors, in any form, adversely affects ruminants’ production by reducing the growth and immunity. Sheep are capable of resisting these stressors, up to a certain level, by modifying their physiological, haematological, biochemical, or a combination of these, in response to thermal environment (Hyder et al., 2017a). The adaptive profiles of sheep were well studied in different farming systems (Kochewad et al., 2018a,b) or seasons (Ribeiro et al., 2016; Rathwa et al., 2017; Banerjee et al., 2015) individually. However, the profile fluctuations in farming systems further depend upon the season. For instance, the adaptation mechanism in extensive sheep may not be similar in winter and summer seasons. As per the collected literature, no studies dealt with the adaptive profile involving different rearing systems with respect to season. Hence, this study was conducted to know the effect of three farming systems with respect to season on body condition scores and physiological, hemato-biochemical, hormonal, enzymatic, and reproductive parameters.

## 2 Materials and methods

### 2.1 Institutional review board statement

The recordings, observations, and blood collection from the sheep were done with approvals of Institute's animal ethics committee (IAEC), Sri Venkateswara Veterinary University (SVVU), Tirupati. The guidelines framed by Committee for the Purpose of Control and Supervision of Experiments on Animals (CPCSEA; IV, section 15(1)) of prevention of cruelty to animals (PETA, 1960) were followed sincerely.

### 2.2 Experimental location

The sheep were maintained at Livestock Research Station (LRS), Sri Venkateswara Veterinary University (SVVU), Palamaner, Chittoor District, Andhra Pradesh. The region is located at 13.2000° N and 78.7500° E and has an average elevation of 683 m (2,244 feet).

### 2.3 Groups, experimental animals and experimental period

**Figure 1:**
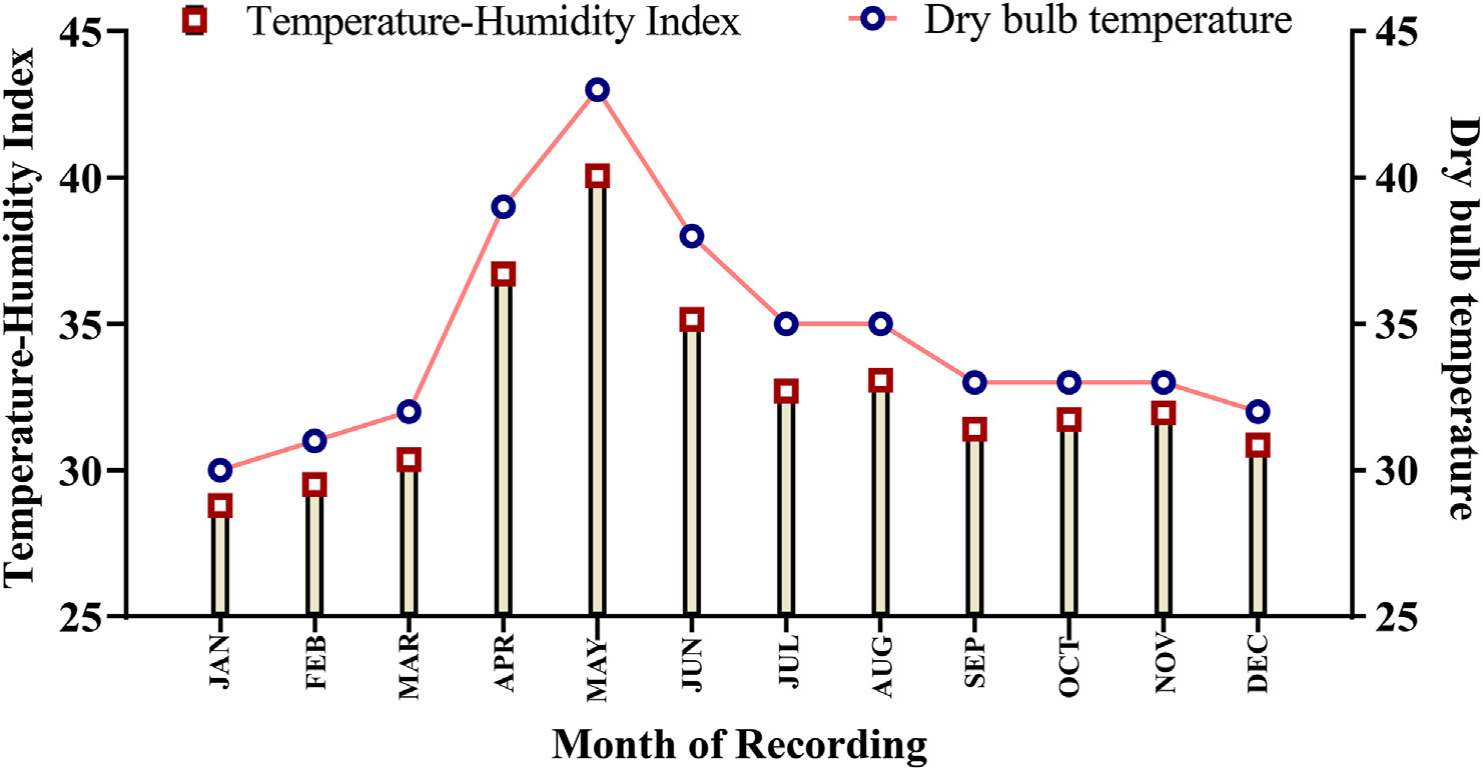
The monthly-wise temperature-humidity index and dry bulb temperature of the farm.

Two parallel trials were conducted to evaluate the parameters under study. In trial I, a flock of 60 ewe-lambs (aged 3 months; S1a File) was allotted to three rearing systems viz. extensive, semi-intensive, and intensive rearing systems and were continuously monitored for puberty, mating, and lambing on daily basis. The breeding was natural for the ewes with a ram: ewe ratio of 1:20. The methodology adopted along with the results of reproductive parameters were presented in our earlier research (Karthik et al., 2021). In trial II, eighteen rams (S1b File) were allotted in a completely randomized design (CRD) to three treatment groups viz., extensive, semi-intensive, and intensive systems (n = 6). The trial II was conducted for 15 months, whereas the ewe-lambs in trial I was monitored for body condition score (BCS) recording for 35 months. The experiment included three seasons (summer, monsoon, and winter) under the Indian conditions. The month-wise average dry bulb temperature and temperature-humidity index (THI), measured during afternoon period (2:00 PM) is presented in Figure 1.

### 2.4 Housing management

The sheep in intensive group were reared with the floor space area of 1m^2^/animal in the covered shed. Fodder and water arrangements were made available in a hygienic way under zero-grazing system. Provisions for proper feeding and watering were made suitably in this group. In extensive system, the animals were moved into night shelter in the covered shed with a floor space of 1m^2^/animal.

### 2.5 Feeding management and health cover

All the experimental sheep under study were properly identified by ear tagging. In extensive system, the sheep were sent for grazing from 8.00 AM to 4.00 PM every day for the entire experimental period. All the experimental animals were offered clean, fresh drinking water, both in shed and outdoor during grazing, during the entire experimental period. The feeding regimen practiced under the three rearing systems is presented in Table 1 while the ingredient and chemical composition of the concentrate mixture fed for semi-intensive and intensive systems are presented in Table 2. Detailed analysis on the type of browse available in the grazing area is presented in Karthik et al. (2021).

**Table 1:** The feeding regimen followed in the current experiment.

Groups	Management
Intensive	concentrate @ 1.5% BW + *adlibitum* green fodder
Semi intensive	6 h grazing + concentrate @1.5% BW
Extensive	8 h grazing

**Table 2:** Ingredients and chemical composition of concentrate mixture.

Composition	Parts
Ingredient composition	
Maize grain	28.00
De Oiled Rice Bran	34.00
Soybean meal	24.00
Groundnut cake	10.00
Urea	1.00
Mineral mixture^1^	2.00
Salt	1.00
Total	100
Chemical composition	
DM	88.50
CP	19.50
NDF	14.87
ADF	6.05

1 Mineral mixture contains vitamin A, 1,000 IU; vitamin D3, 250 IU; vitamin E, 7.5 IU; Ca, 1 g; P, 0.3 g; Cu, 3.25 mg; Fe, 0.2 mg; Zn, 15; mn, 3.25 mg; I, 0.2 mg; Se, 0.1 mg.

All the experimental animals were dewormed before starting the experimental trial with broad-spectrum anthelmintic (I.P. 500 mg Niclosamide and 150 mg Albendazole; Vet India Pvt. Ltd.). Deticking was done twice during the experimental period for the control of external parasites with amitraz dip concentrate (I.P. 12.5% w/v; Virbac India Pvt. Ltd.). All the animals were vaccinated against FMD (Indian Immunologicals Pvt. Ltd., India), Enterotoxaemia (Brilliant biopharma Pvt. Ltd., India), Blue tongue (Indian Immunologicals Pvt. Ltd., India), and PPR (Hester Biosciences Ltd., India). Hygienic surroundings were maintained throughout the experimental period.

### 2.6 Sample collection and recordings

The ewes were monitored for body condition scoring (BCS) during puberty, mating, 2 weeks pre-lambing and 2-, 4-, 8-, and 12-weeks post-lambing. The three seasons used for collection of the samples were winter (November to January), summer (March to May), and monsoon (July to September) seasons. The hours of recording for physiological parameters include 2:00 AM, 8:00 AM, 2:00 PM, and 8:00 PM during each day of collection; however, the hematological parameters, biochemical parameters, and oxidative stress parameters were evaluated from blood collected at 8:00 AM and 2:00 PM from the rams during three consecutive days of each season. A body condition scoring (BCS) of 1–5 scaling was used with an interval of 1.0 unit (Maurya et al., 2008).

The rectal temperature (°F) was recorded with the help of a digital thermometer (Accusure MT-1027) by keeping it in contact with rectal mucosa for one min. The count of arterial pulse per minute was recorded by palpating at femoral artery by trained fingertips. The respiration rate of the experimental sheep was recorded by visual observations of inward and outward abdominal movements. One inward and outward movement was counted as one respiration and respiration rate was expressed in numbers per minute. For haemato-biochemical studies, a total of 5.0 ml of blood was collected from jugular vein of the experimental sheep. The blood was collected at 8:00 AM and 2:00 PM during the three seasons using a sterilized plastic syringe and needle and EDTA coated vials. Out of the collected blood, 4.0 ml was subjected for plasma separation for biochemical analysis and remaining 1.0 ml was used for hematological studies. Semen was collected from six rams under each system twice a day by using an estrus ewe as dummy with a standard artificial vagina after allowing two false mounts each time. The artificial vagina was prepared by assembling an outer cylinder (disinfected soft rubber of 20 cm length and 5 cm width), latex cone (6″), smooth latex liner (12″), insulation bag, graduated collection tube, and warm water at 45 °C. Soon after collection, the semen collection tubes were labelled and transferred to a water bath at 34 °C and immediately evaluated for sperm count, progressive sperm motility, plasma membrane integrity, minor abnormalities, major abnormalities, and total abnormalities.

### 2.7 Laboratory analysis and calculations

The hemoglobin concentration (g/dl) was estimated according to the cyanomethemoglobin method using Sahli's haemoglobinometer (M/S Singhla scientific industries, Haryana, India). The packed cell volume (PCV) of the collected blood sample was measured by the procedure given by Wintrobe method (Wintrobe, 1976). Total erythrocyte count was estimated by haemocytometer (M/S Rohem instruments Pvt. Ltd., Maharashtra, India) as per the protocol given by Jain (1993).

Total leucocytes count (TLC) was estimated by haemocytometer as per the protocol of Jain (1993).

Differential leukocyte count is enumerated by using leishmans stained blood smear based on the procedure mentioned by Jain (1993).

The serum biochemical parameters viz. glucose, total protein, albumin, globulin, cholesterol, blood urea nitrogen (BUN), creatinine, uric acid, aspartate amino transferase (AST), alanine amino transferase (ALT), calcium, and phosphorous were calculated by using the standard kits ((M/s. ERBA diagnostics Mannheim GmbH). The triiodothyronine (T3), thyroxine (T4), and cortisol were estimated with CLIA (Chemiluminescence Immunoassay) kits with different detectable ranges. The superoxide dismutase (SOD) was estimated by colorimetric method using activity assay kit (Elabscience®) with a detection range of 2.03–155 U/ml, intra-assay CV of 4.0% and inter-assay CV of 7.2%. The malondialdehyde (MDA) activity was assayed as per the thiobarbituric acid method by using commercial kits (Elabscience®) with a detection range of 2.92–200 μmol/L, intra-assay CV of 4.1% and inter-assay CV of 7.2%. The glutathione activity (GPx) was measured by colorimetric method using activity assay kit (Elabscience®) with a detection range of 12.65–387.0 U, intra-assay CV of 4.9% and inter-assay CV of 9.3%. The catalase activity was estimated by using commercial kit (Elabscience®) with a detection range of 1.12–150.0 U/ml, intra-assay CV of 3.9% and inter-assay CV of 7.7%.

### 2.8 Reproductive parameters of rams

The dartos muscle extension was measured as per the method described by Maurya et al. (2017). The sweating rate (g/m^2^/h) was measured as per the protocol of Schleger and Turner (1965). The sperm concentration was determined by counting the total number of sperms in Neubauer counting chamber after diluting semen with 1:200 with a formaldehyde-based diluting fluid. The progressive sperm motility (%) was measured by observing under 450x magnification. The sperm motility was observed under high power at 450x magnification and expressed in percentage of progressively (0–100) motile sperms.

Plasma membrane integrity of the sperms was assessed by hyposmotic swelling test, as provided by Jeyendran et al. (1984). The sperm abnormalities (%) were outlined and categorized according to the protocol of Bloom (1973). All the sperms were counted for the presence of head, mid piece, and tail abnormalities. Head abnormalities constitute macro, micro, detached, and pear-shaped heads. The midpiece abnormalities include bent, coiled and kinked neck mid pieces. Tail abnomalities were broken, coiled, and kinked tails.

### 2.9 Statistical analysis

The values of physiological, hamato-biochemical, oxidative, and male reproductive parameters were tested for normal distribution by using Kolmogorov-Smirnov test. The data of physiological, haemato-biochemical, oxidative, and male reproductive parameters were analyzed through GLM multivariate procedure. The sheep was used as a random effect and the season and interactions among farming system (FS) and season (S) were used as fixed effects. The initial values of body weights were different among the three groups; hence, they were included in the model as covariates. Post hoc analysis, wherever necessary, is performed by adjusting the data as per the Bonferroni corrections and Tukey's HSD. The recordings at different hours were pooled and the values were presented as means with standard error of means. Because of the variations in conception percent and pregnancy rate, the sample size of BCS differed between the three groups. The BCS (ordinal data) with reference to feeding system and season were analyzed for differences according to Kruskal Wallis H test. The *P* values less than 0.05 is considered as significant and those between 0.05 and 0.1 was considered as a trend. Entire statistical analysis was performed by using SPSS version 23.0 (IBM Corp, 2015). To know the adaptive profile of sheep, the data pertaining to intensive (no stress) and extensive systems (heat, nutritional, and walking stress) were subjected to principal component analysis by using ‘prcomp’ function in R version 3.6.3 (RStudio Team, 2020).

## 3 Results

### 3.1 Body condition scoring of ewes

**Figure 2:**
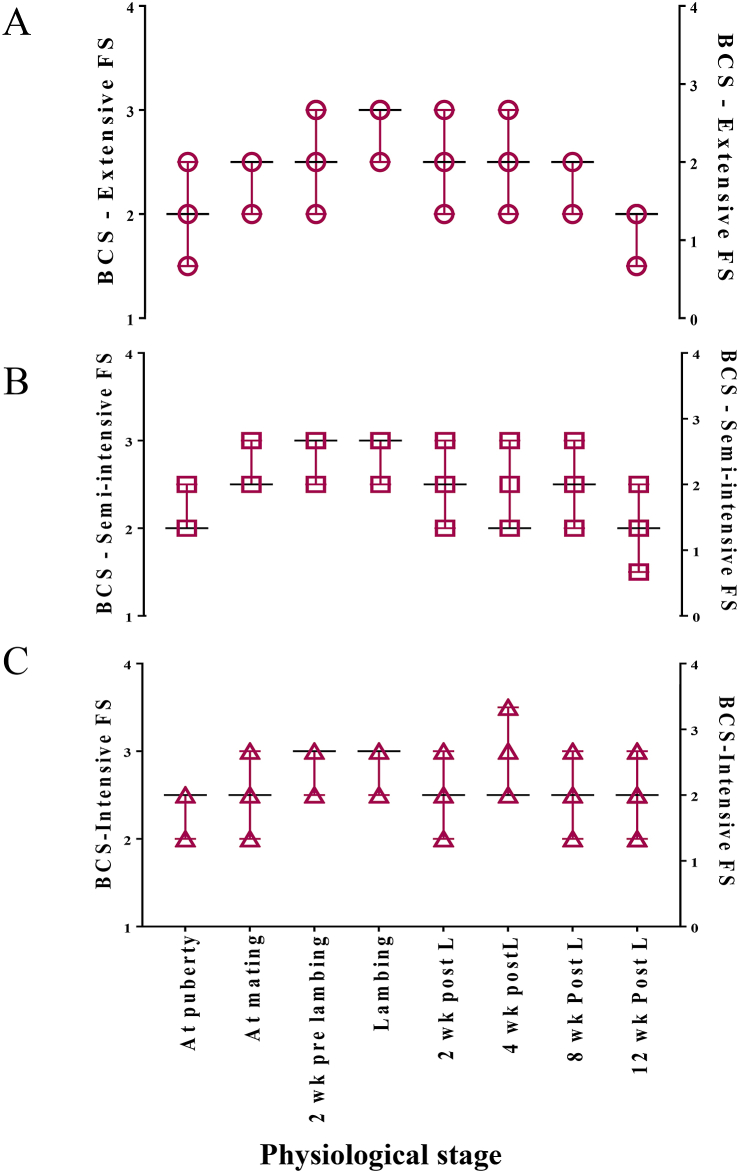
Body condition score of ewes with reference to farming system and physiological stage. (A) BCS in extensive farming system (B) BCS in semi-intensive farming system and (C) BCS in intensive farming system.

The median values and Kruskal-Wallis (KW) ranks of the BCS of sheep reared under three rearing systems are presented in Table 3. The scores on the scale of 0–5 of the ewes with reference to various physiological stages and rearing systems are depicted in Figure 2. No significant changes were observed for BCS at all the measured periods, except for 12 wk-post-lambing, which was higher (P < 0.001) in intensive FS. Further, the KW ranks were higher for ewes reared under intensive FS followed by semi-intensive and extensive systems.

**Table 3:** Body condition scoring of the ewes under various physiological stages with reference to different rearing systems.

Period (months)	Rearing System			SEM	P Value
	EXT	SI	INT		
At puberty	2.0 (16.15)	2.0 (23.46)	2.5 (24.25)	0.07	0.086
At mating	2.0 (14.42)	2.5 (25.19)	2.5 (24.25)	0.07	0.011
2 wk pre-lambing	2.5 (17.65)	3.0 (22.03)	3.0 (24.69)	0.07	0.236
At lambing	3.0 (19.65)	3.0 (21.27)	3.0 (23.19)	0.05	0.441
2 wk post-lambing	2.5 (20.08)	2.5 (21.50)	2.5 (22.66)	0.07	0.751
4 wk post-lambing	2.5 (17.31)	2.5 (20.08)	2.5 (26.06)	0.10	0.091
8 wk Post-lambing	2.5 (20.69)	2.5 (21.15)	2.5 (26.44)	0.09	0.906
12 wk Post-lambing	2.0 (14.50)	2.0 (16.34)	2.5 (31.38)	0.08	<0.001

The values presented were medians with mean Kruskal-Wallis ranks in parenthesis.

Ext - Extensive, SI - Semi intensive, I - Intensive, SEM - Standard error of mean, *P* Val - *P* values.

### 3.2 Physiological parameters

The physiological parameters with reference to different rearing systems and season of recording are presented in Table 4. The sheep reared in extensive and semi-intensive system exhibited higher temperature, pulse rate, and respiratory rate. Similarly, the physiological parameters were higher in summer season. No interactions were noticed among the rearing season and time or season.

**Table 4:** Physiological parameters of sheep with reference to different rearing systems and season.

Parameter	Rearing system			Season			SEM	*P* Val		
	EXT	SI	INT	WIN	MON	SUM		RS	S	RS×S
TEMP (°F)	102.1^b^	101.98^b^	101.62^a^	101.9^a^	101.65^a^	102.18^b^	0.05	<0.001	<0.001	0.792
PR (no./min)	88.39^b^	86.75^b^	83.57^a^	86.54^ab^	83.86^a^	88.31^b^	0.54	0.001	0.004	0.815
RR (no./min)	26.50^b^	25.44^b^	23.61^a^	25.4^b^	22.15^a^	28.00^c^	0.34	<0.001	<0.001	0.202

^abc^Rows bearing different superscripts differ significantly.

TEMP – Rectal temperature, PR – Pulse rate, RR – Respiratory rate, Ext - Extensive, SI – Semi intensive, I - Intensive, SEM – Standard error of mean, T – Time of recording, RS – Rearing system, S – Season, *P* Val - *P* values.

### 3.3 Haematological parameters

Haematological parameters of different rearing systems with respect to season and hour of recording is shown in Table 5. Extensive and semi intensive rearing decreased the haemoglobin, PCV, and RBC, while increasing the WBC without affecting the MVC, MCH, MCHC, and the differential leucocyte. The percent haemoglobin and RBC count were higher in winter than summer months, whereas WBC count followed an exactly opposite pattern. No other parameters showed diurnal variation, except haemoglobin, WBC, MCH, and MCHC. No interactions were noticed for rearing system vs. season or time and time and season, except a tendency (P = 0.084) for time and season for haemoglobin percent.

**Table 5:** Haematological parameters with reference to different rearing systems and seasons.

Parameter	Rearing System			Season		SEM	*P* Val		
	EXT	SI	INT	WIN	SUM		RS	S	RS×S
Haemoglobin (g/dl)	11.56^a^	11.61^a^	12.88^b^	12.30	11.74	0.15	<0.001	0.015	0.391
PCV (%)	29.42^a^	29.63^a^	31.46^b^	30.61	29.72	0.30	0.009	0.127	0.905
RBC (×10^6^μl)	9.86^a^	9.92^a^	10.44^b^	10.24	9.91	0.06	<0.001	0.001	0.659
WBC (×10^3^μl)	9.10^b^	9.13^b^	8.35^a^	8.62	9.10	0.08	<0.001	<0.001	0.904
MVC (fl)	29.83	29.87	30.26	29.94	30.03	0.31	0.840	0.898	0.733
MCH (pg)	11.72	11.71	12.39	12.03	11.85	0.15	0.083	0.506	0.674
MCHC (g/dl)	39.37	39.36	41.22	40.39	39.57	0.52	0.239	0.427	0.457
Neutrophils (%)	36.04	36.21	35.21	35.94	35.69	0.25	0.248	0.631	0.523
Lymphocytes (%)	57.75	57.21	58.13	57.64	57.75	0.36	0.614	0.884	0.805
Monocytes (%)	2.63	2.50	2.54	2.58	2.53	0.07	0.806	0.727	0.970
Eosinophils (%)	2.75	2.88	2.96	2.86	2.86	0.11	0.771	1.000	0.884
Basophils (%)	0.83	0.75	0.71	0.97	0.56	0.11	0.906	0.080	0.959

^abc^ Rows bearing different superscripts differ significantly.

PCV - Packed cell volume (%), RBC - Red blood cells, WBC - White blood cells, MVC - Mean corpuscular volume, MCH - Mean corpuscular hemoglobin, MCHC - Mean corpuscular hemoglobin concentration, Ext - Extensive, SI - Semi intensive, INT - Intensive, SEM - Standard error of mean, T - Time of recording, RS - Rearing system, S - Season, *P* Val - *P* values.

### 3.4 Biochemical parameters

The biochemical parameters altered by rearing systems and season are presented in Table 6. The sheep raised in intensive system showed higher glucose, total protein, albumin, cholesterol, T3, T4, calcium, and phosphorus levels. However, the globulin, creatinine, uric acid, AST, and ALT were elevated in extensive and semi-intensive systems. Summer month noticed higher levels of cholesterol, BUN, creatinine, uric acid, AST, ALT, and cortisol. No interactions were noticed for any of the parameters, except for cortisol, which showed significant interactions for rearing system vs season.

**Table 6:** Biochemical and hormonal parameters with reference to different rearing systems and seasons.

Parameter	Rearing System			Season		SEM	*P* Value		
	EXT	SI	INT	WIN	SUM		RS	S	RS×S
Biochemical profile									
Glucose (mg/dL)	45.00^a^	50.92^b^	59.50^c^	52.72	50.89	0.85	<0.001	0.060	0.970
Total protein (g/dL)	6.79^a^	7.05^a^	7.63^b^	7.33	6.98	0.06	<0.001	<0.001	0.814
Albumin (g/dL)	3.01^a^	3.85^b^	4.34^c^	4.01	3.45	0.08	<0.001	<0.001	0.761
Globulin (g/dL)	3.78^b^	3.20^a^	3.29^a^	3.32	3.53	0.06	<0.001	0.065	0.557
Cholesterol	48.54^a^	51.33^a^	82.79^b^	62.56	59.22	1.94	<0.001	0.003	0.910
BUN (mg/dL)	18.54	19.08	18.75	17.72	19.86	0.32	0.755	0.009	0.935
Creatinine (g/dL)	2.12^b^	2.08^a^	1.56^a^	1.76	2.08	0.05	<0.001	<0.001	0.564
Uric acid (mg/dL)	0.37^c^	0.22^b^	0.15^a^	0.21	0.28	0.02	<0.001	<0.001	0.487
AST (IU/L)	110.38^b^	102.79^b^	85.00^a^	89.69	109.08	2.15	<0.001	<0.001	0.224
ALT (IU/L)	40.21^c^	35.54^b^	31.58^a^	33.06	38.50	0.83	<0.001	<0.001	0.858
Calcium (mg/dL)	7.57^a^	7.87^a^	8.66^b^	8.21	7.85	0.10	<0.001	0.048	0.456
Phosphorous (mg/dL)	4.28^a^	4.48^a^	5.46^b^	4.90	4.58	0.08	<0.001	0.006	0.772
Hormonal profile									
T3 (ng/mL)	1.55^a^	2.04^b^	2.89^c^	2.25	2.07	0.08	<0.001	0.018	0.250
T4 (ng/mL)	45.75^a^	50.38^b^	56.58^c^	53.42	48.39	0.75	<0.001	<0.001	0.140
Cortisol (ng/mL)	9.55^c^	8.05^b^	6.41^a^	6.06	9.95	0.32	<0.001	<0.001	0.034

^abc^ Rows bearing different superscripts differ significantly.

BUN - Blood urea nitrogen, AST - Aspartate amino transferase, ALT - Alanine amino transferase, T_3_ - Triiodothyronine, T4 - Thyroxine, Ext - Extensive, SI - Semi intensive, I - Intensive, SEM - Standard error of mean, T - Time of recording, RS - Rearing system, S - Season, *P* Val - *P* values.

### 3.5 Oxidative stress parameters

The oxidative stress parameters of the sheep maintained under different rearing systems are presented in Table 7. The sheep reared under extensive system showed higher values of SOD, MDA, GPx, and catalase followed by semi-intensive and intensive systems. No interactions were noticed among all oxidative stress parameters.

**Table 7:** Oxidative stress parameters with reference to different rearing systems and seasons.

Parameter	Rearing System			Season		SEM	*P* Val		
	EXT	SI	INT	WIN	SUM		RS	S	RS×S
SOD (U/ml)	96.06^c^	85.00^b^	74.75^a^	79.80	90.74	2.03	<0.001	<0.001	0.101
MDA (μmol/l)	4.10^c^	3.77^b^	3.22^a^	3.54	3.86	0.07	<0.001	0.003	0.913
GPx (U/ml)	117.46^c^	111.25^b^	97.54^a^	105.06	112.44	1.31	<0.001	<0.001	0.842
Catalase (U/ml)	3.79^c^	3.36^b^	2.85^a^	3.19	3.47	0.10	<0.001	0.018	0.728

^abc^ Rows bearing different superscripts differ significantly.

SOD – Superoxide dismutase (U/ml), MDA – Malondialdehyde (μmol/l), GPx – Glutathione peroxidase (U/ml), Ext - Extensive, SI – Semi intensive, I – Intensive, SEM – Standard error of mean, T – Time of recording, RS – Rearing system, S – Season, *P* Val – *P* values.

### 3.6 Reproductive parameters of rams

The reproductive parameters of rams with reference to rearing system and season are presented in Table 8. The dartos muscle extension (cm) and scrotum sweating rate (g/m^2^/h) were higher (P < 0.001) in the sheep reared under extensive system and during summer season. The seminal parameters viz., total sperm count, progressive sperm motility, and plasma membrane integrity were higher in intensively reared sheep. Further, the percent of sperm abnormalities, including both major and minor abnormalities, were lesser in intensive system. No seasonal influences were reported on all the seminal parameters studied.

**Table 8:** Male reproductive parameters at different rearing systems with respect to season.

Parameter	Rearing system			Season		SEM	P Value		
	EXT	SI	INT	WIN	SUM		RS	S	RS×S
Dartos muscle extension (cm)	14.51^c^	12.38^b^	10.72^a^	10.72	14.35	0.12	<0.001	0.041	0.032
Scrotum sweating rate (g/m^2^/h)	601.50^c^	521.42^b^	311.0^a^	349.34	606.6	8.64	<0.001	<0.001	0.025
Sperm count (×10^9^spermatozoa)	3.21^a^	3.42^ab^	3.75^b^	3.47	3.44	0.07	0.004	0.823	0.931
Progressive sperm motility (%)	69.83^a^	74.83^b^	75.33^b^	73.61	73.06	0.74	0.003	0.676	0.993
Plasma membrane integrity (%)	62.42^a^	66.58^b^	67.92^b^	66.00	65.28	0.62	<0.001	0.484	0.988
Minor abnormalities (%)	3.98^b^	3.26^b^	3.08^a^	3.41	3.47	0.09	<0.001	0.597	0.994
Major abnormalities (%)	3.74^b^	3.49^b^	2.99^a^	3.32	3.49	0.09	0.002	0.284	0.895
Total abnormalities (%)	7.72^c^	6.75^b^	6.06^a^	6.72	6.97	0.15	<0.001	0.230	0.903

^abc^ Rows bearing different superscripts differ significantly.

Ext - Extensive, SI – Semi intensive, I – Intensive, SEM – Standard error of mean, T – Time of recording, RS – Rearing system, S – Season, *P* Val – *P* values.

### 3.7 Principal component analysis and vector plot

**Figure 3:**
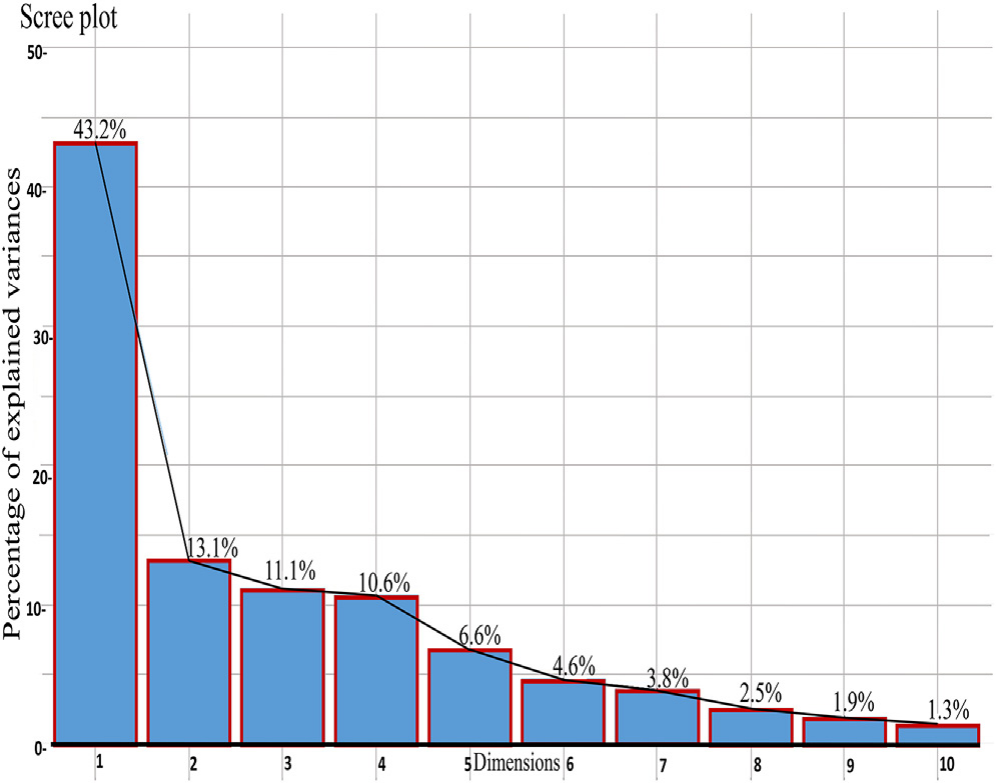
Scree plot showing Eigen value variance percent of various dimensions.

**Figure 4:**
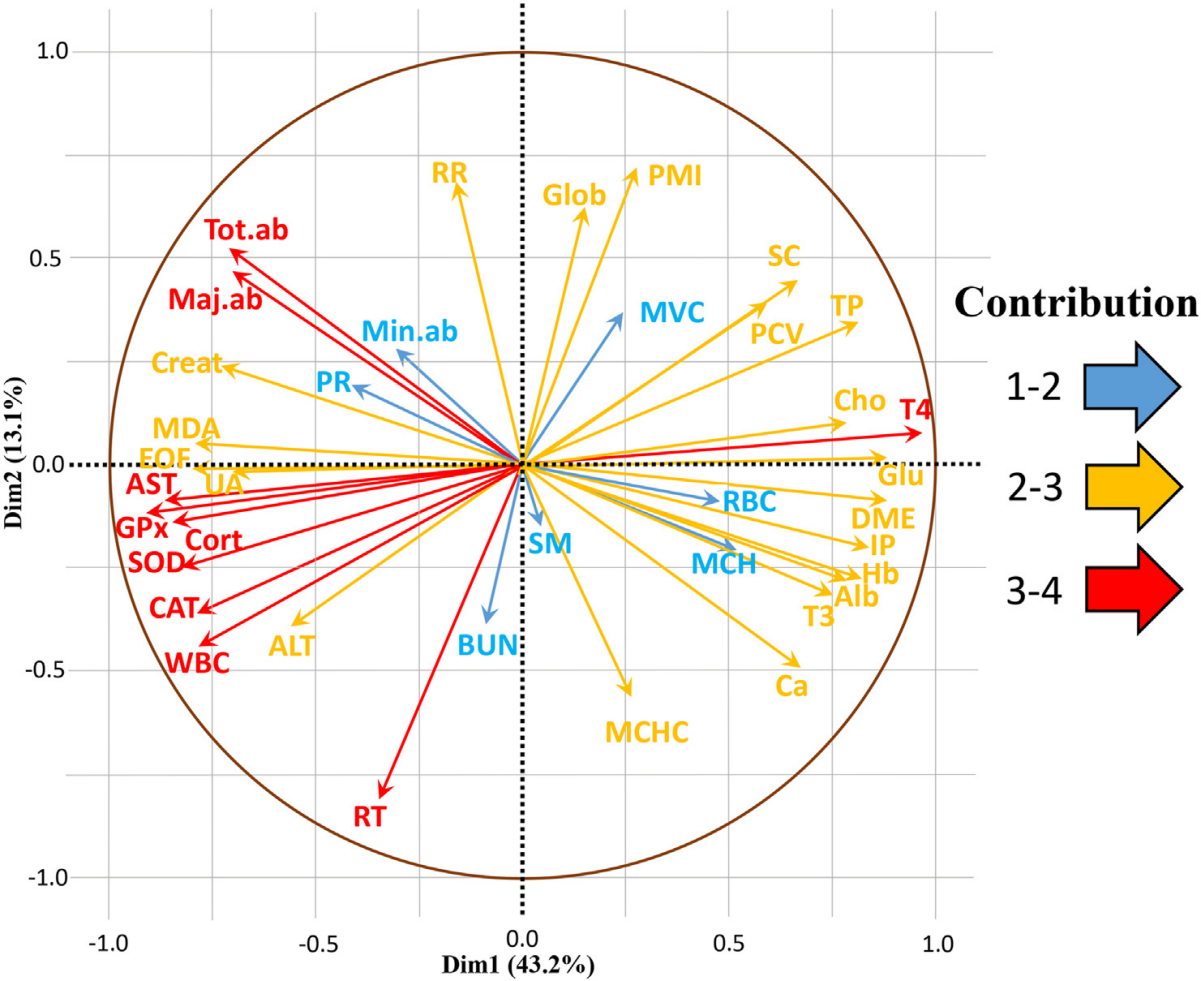
Vector plot showing variance contributors of sheep exposed to multiple stressors (Intensive vs. Extensive farming system). Tot. ab, total abnormalities; Maj. Ab, major abnormalities; Min. ab, minor abnormalities; Creat, Creatinine; PR, pulse rate; MDA, malondialdehyde; EOF, erythrocyte osmotic fragility; AST, aspartate transaminase; GPx, glutathione peroxidase; SOD, superoxide dismutase; CAT, catalase; WBC, white blood cells; ALT, alanine aminotransferase; RT, rectal temperature; BUN, blood urea nitrogen; SM, sperm motility; MCHC, mean corpuscular hemoglobin concentration; Ca, calcium; MCH, mean corpuscular hemoglobin; T3, Triiodo thyronine; Alb, albumin; Hb, hemoglobin; DME, dartos muscle extension; RBC, red blood cell; Glu, glucose; T4, Thyroxine; Cho, Cholesterol; TP, total protein; PCV, packed cell volume; SC, sperm count; MVC, mean corpuscular volume; PMI, plasma membrane integrity; Glob, Globulin; RR, Respiratory rate.

The scree plot and vector plots are presented in Figures 3 and 4, respectively. Scree plot revealed the variance percent of 43.21, 13.12, and 11.15 in dimensions 1, 2, and 3, respectively, with corresponding Eigenvalues of 15.55, 4.7, and 4.01. The vector plot showed that the AST, GPx, Cortisol, SOD, Catalase, WBC, PR, T4, total abnormalities, and major abnormalities were the major contributors for adapting during combined stressors (heat and nutritional). However, the parameters such as RR, MVC, SM, BUN, MCH, RBC, and Min. ab. were not readily variable to the stressors.

## 4 Discussion

### 4.1 Body condition scoring of ewes

Because of the easily calculated complexion and its linear relationship with body energy reserves, BCS could be considered as a direct indicator of energy reserves mobilization (Reddy et al., 2018). In the present study, the management system did not significantly influence the median values of body condition score during all the recorded periods, except 12 wk post-lambing. However, analyzing the Kruskal-Wallis ranks revealed a wide difference between the three farming systems with higher ranks in intensively reared ewes. Diversion of nutrients for grazing activities and exposure to high ambient temperature for a period of 6 or 8 h might have caused loss of body reserves in extensive and semi-intensive systems, consequently lowering the BCS ranks. Ewes with low BCS are known to prone to stillbirths, prenatal and neonatal mortality, low birth weights and decreased lamb survival percent (Gardner et al., 2006). The BCS of all the ewes under experiment ranged from 2.0 - 3.0, indicating the ideal muscle thickness and body fat reserves as per the physiological condition of the ewe.

The BCS ranks of ewes during puberty, mating, lambing, and post-lambing undoubtedly state that the intensively reared ewes are better in lambing performance and post-lambing energy gain for next mating. Within the group, the BCS of ewes depends upon the energy requirement, which further relies on metabolic and physiological status. The appetite, dry matter intake, energy balance, and reproductive function are highly correlated to the alterations in energy uptake.

### 4.2 Physiological parameters

The physiological parameters viz., rectal temperature, pulse rate, and respiratory rate of sheep are pertinent indicators of their comfort during heat stress and related environment (Hyder et al., 2017b). The primary adaptive mechanism of homeotherms during heat stress and raised core temperature is the cooling mechanism by rapid respiration and increased pulse rate. The sheep prone to heat stress viz. those reared in extensive management system or summer season showed higher rectal temperature, pulse rate, and respiratory rate. Several authors confirmed the heat stress-induced alteration in physiological parameters earlier (Rathwa et al., 2017; Wojtas et al., 2014; Ramana et al., 2013; Rashid et al., 2013). According to Habibu et al. (2017a), the increased rectal temperature during heat stress is a physiological response due to the increased oxygen delivery to the lungs, thus improving the heat production and elevated body temperature. The increased pulse rate may augment the blood flow under the body surface, therefore dissipating more heat through conduction, convection, radiation, and water diffusion from skin (Adedeji, 2012; Habibu et al., 2014). The higher pulse rate might result from rapid heartbeat and metabolism due to grazing for long distances (Kochewad et al., 2017). The increased respiratory rate could be attributed to the long walks in semi-intensive and extensive systems (Kochewad et al., 2018a, b).

### 4.3 Hematological parameters

Sheep sent for grazing exhibited lower Hb, PCV and RBC contents. These results are in line with those reported elsewhere (Habibu et al., 2017b; Banerjee et al., 2014; Singh et al., 2016). Sejian et al. (2010) reported lower Hb levels in the sheep maintained on a low plane of nutrition and depleted nutrient resources. The heat stress may precipitate haemoglobin molecules in the erythrocytes, resulting in decreased Hb concentration (Giulivi et al., 1994). Extensive sheep exhibited a decreased PCV percent; however, Kochewad et al. (2017) reported a higher PCV in sheep allotted for grazing rather than stall-feeding. The authors attributed the increased PCV to hemo-concentration developed due to asphyxia and dehydration, leading to release of erythrocytes concentrated in spleen. Nevertheless, the ample supply of clean drinking water to the grazing sheep throughout the experimental period might have combated the hemo-concentration and altered the results in our study.

The heat stress increased WBC count of sheep during extensive rearing and summer season. Likewise, few reports exist on elevated levels of leucocytes in heat-stressed goats (Habibu et al., 2017b). Heat stress did not affect the proportion of individual leucocyte fractions viz., neutrophils, lymphocytes, monocytes, eosinophils, and basophils. On the contrary, Habibu et al. (2017b) revealed a higher neutrophils count followed by lymphocytes (OmranFayza et al., 2011). Neither rearing system nor season altered the MVC, MCH, and MCHC. In contrast, few authors reported either increased (Abdelatif et al., 2009) or decreased (Ribeiro et al., 2018; Mazzullo et al., 2014) MVC in small ruminants under heat stress.

### 4.4 Biochemical parameters

No seasonal influence was noticed for serum glucose levels; however, the sheep reared in extensive system revealed decreased serum glucose concentration. Similarly, few authors found reduced glucose concentration in sheep grazed for 8 h per day (Kulkarni et al., 2010; Kochewad et al., 2018b). The phenomenon indicates that depleted nutrition, but not the heat load, affect glucose concentration. In extensive system, the adaptive mechanism of fermentative metabolism of sheep might have increased the glucose utilization for body functions, ultimately decreasing their serum levels (Hyder et al., 2017b). Heat stress elevates plasma insulin concentrations in the bloodstream, essential for glucose disposal in peripheral tissues (Min et al., 2015). The less availability of substrate for glucose formation in extensive system is another reason for the low serum glucose levels.

The total protein and albumin contents were higher (P < 0.001) in the sheep under intensive rearing and winter season, while the globulin contents followed opposite pattern. The higher serum total protein in intensively reared sheep could result from greater converting efficiency of non-protein nitrogenous substances into amino acids and protein (Reddy et al., 2019a, b). The intensively reared sheep could have better rumen microbial count to synthesize proteins from available non-protein nitrogen contents, as the diets contain urea.

The two tested independent factors viz., season and rearing system affected serum levels of AST and ALT, which were increased in heat-stressed sheep. In general, the increased AST levels are related to liver and muscle-related diseases, and the higher ALT levels are due to the liver disorders alone. However, in the present study, these results could be attributed to the increased gluconeogenesis from protein sources, thereby stressing the liver (Kochewad et al., 2018b). According to Weigand et al. (2007), higher serum levels of AST or ALT in any species are related to their lower heat tolerance ability. Similar results of higher AST and ALT levels were reported in Nellore sheep (Nejad and Sung, 2017) and Deccani sheep (Kochewad et al., 2017) maintained under extensive grazing systems. The circulating concentrations of T3 and T4 levels were lower in extensive or semi-intensive systems and summer season. These results are in corroboration with the results of the earlier research (Indu et al., 2014; Ribeiro et al., 2016; Habibu et al., 2017b). The heat stress is known to affect hypothalamic-pituitary-thyroid axis to decrease the production of thyroid hormones thereby preventing metabolic heat production to cope with elevated ambient temperature (Aleena et al., 2016).

The significant interaction between rearing system and season for serum cortisol levels clearly indicate that the heat stress impact was not the same in all the three rearing systems during summer periods. Increased heat load stimulus from the thermoreceptors of skin rises the plasma cortisol levels by activating the Adreno-corticotrophin (ACTH) releasing mechanism (Yang and Zhang, 2004). The raised cortisol level aids in vasodilation to facilitate heat loss. The higher calcium and phosphorus contents of intensively reared sheep's serum could be directly related to high plane of nutrition viz. concentrate and fodder feeding. The decreased serum calcium and phosphorus levels during heat stress were well-documented (Allahverdi et al., 2013; Ayo et al., 2011).

### 4.5 Oxidative stress parameters

Along the year, the serum levels of SOD, MDA, GP_X_, and catalase enzymes varied with the highest quantities in summer season. The plasma oxidative stress parameters viz., SOD, MDA, GPx, and catalase were higher (p < 0.001) in extensively reared sheep. Heat stress predisposes oxidative stress by increasing the production of oxygen free radicals (Ghosh et al., 2013). The higher concentration of SOD, MDA, GPx, and catalase observed in extensive sheep, especially during summer periods, might be related to the heat stress condition (Chaudhary et al., 2015).

### 4.6 Reproductive parameters of rams

Relaxation of the dartos muscle in summer season and extensive and semi-intensive rearing systems is perhaps the most significant observation of the trial. The extension of tunica dartos muscle occurs as much as possible to dissipate heat from the testis (Marai et al., 2007). The higher scrotum sweating rate (g/m^2^/h) in extensive sheep reveal that the SSR is an important adaptive measure for rams to counter the heat stress. The functional and morphological dynamics of scrotal sweat glands are crucial for efficient latent heat dissipation, especially during heat stress (Ferreira et al., 2009).

The semen collected from semi-intensively reared sheep showed normal sperm count, progressive sperm motility, and plasma membrane integrity; however, the sperm abnormalities were significantly higher than intensively reared sheep. The sheep reared under semi-intensive farming system were predicted to experience heat stress alone; however, the sheep raised under extensive system were prejudiced to subject for both heat and nutrition stress. Hence, it is clear that the decreased sperm count, motility, and plasma membrane integrity could be related to nutritional stress. The decreased calcium and potassium ion exchange in nutritional stress animals may affect the spermatogenesis and the excitation and differentiation of germinal (seminiferous) epithelium, consequently affecting the sperm count, motility and plasma membrane integrity (Hagiwara and Kawa, 1984). Particularly, the intracellular calcium ion in spermatozoa is important in regulating the flagellar motility of sperm and fusion of vesicle.

The higher sperm abnormalities’ count in heat stressed-sheep might be due to the slight alterations in the seminiferous epithelium (Thundathil et al., 2012; Moretti et al., 2014). The time and temperature of exposure are two important factors causing testicular degeneration in heat-stressed sheep (Thundathil et al., 2012). These results are in lieu with Kahwage et al. (2018), who reported a slight increase in total and major abnormalities because of the intense environmental heat. However, the authors also noticed that the morphological abnormalities were within the maximum acceptable threshold count. The study revealed absence of thermal stress effect on semen volume and count. Even in the event of increased rectal temperature, it is imperative to note that the thermal gradient between the distal and proximal poles of testis will be maintained because of the thermoregulatory mechanisms (Alves et al., 2016; Kahwage et al., 2018). No seasonal effects were noticed on the seminal parameters. Similarly, Aguiar et al. (2013) reported unaltered sperm motility, vigor, volume, and concentration in any of the seasons.

### 4.7 Principal component analysis and vector plot

The vector lengths were higher for AST, GPx, cortisol, SOD, catalase, WBC, RT, T4, Tot. ab., and maj. ab. revealing them as highly variable parameters in adaptation. Rectal temperature (RT) was the most sensitive indicator among the physiological responses, as mentioned by a few authors. (Polsky and Von Keyserlingk, 2017; Ferreira et al., 2019). Activation of hypothalamic-pituitary-adrenal axis is one of the first physiological responses on exposing animal to stressful conditions, causing an immediate alteration in serum concentrations of T3 and T4 (Façanha et al., 2013). According to Rathwa et al. (2017), BUN is hypothesized to alter more readily during stress; however, the contribution of BUN for the adaptive profile is low (Rathwa et al., 2017). The urea content in concentrate fed to intensive sheep could have increased the BUN concentration similar to that of stressed sheep (extensive sheep), therefore countering the fluctuations. Among the parameters representing blood mechanism, WBC was the most variable component, which might be related to their short life span and the low plane of nutrition (Pietrocola et al., 2017). The seasonal changes in the adaptive profile displays true thermal resilience of Nellore sheep in resisting the challenges of temperature fluctuations (Reddy et al., 2019a, b).

## 5 Conclusion and recommendations

Subjecting the Nellore sheep to nutritional, heat, and walking stressors compromised the energy status and modified physiological, hemato-biochemical, hormonal, enzymatic, and reproductive parameters as a measure of adaptation. As in semi-intensive system, experiencing heat stress alone did not affect sperm count, motility, and plasma membrane integrity, revealing the mechanisms of acclimation by relaxing the tunica dartos muscle and increasing scrotum-sweating rate. The study projects Nellore sheep breed as a climate-resilient breed, which can be used for crossbreeding with exotic sheep to sustain tropical Indian conditions. Further, the study concludes that the aspartate aminotransferase, glutathione peroxidase, cortisol, superoxide dismutase, catalase, white blood cells, rectal temperature, thyroxine, total abnormalities, and major abnormalities were the highly variable parameters for adaptation to heat, nutritional, and walking stressors. Nevertheless, the current study did not quantify the extent of stress in the extensive and semi-intensive sheep. We believe our work could form a basis for stress-related works in Nellore sheep, and our investigations into this area are still ongoing.

## Declarations

### Author contribution statement

D. Karthik: Conceived and designed the experiments; Performed the experiments; Analyzed and interpreted the data.

J. Suresh; Y. Ravindra Reddy; G.R.K. Sharma; J. V. Ramana: Conceived and designed the experiments.

G. Gangaraju: Contributed reagents, materials, analysis tools or data.

P. Pandu Ranga Reddy; Y. Pradeep Kumar Reddy; D. Yasaswini: Analyzed and interpreted the data.

M.J. Adegbeye: Wrote the paper.

P. Ravi Kanth Reddy: Performed the experiments; Analyzed and interpreted the data; Wrote the paper.

### Funding statement

This research did not receive any specific grant from funding agencies in the public, commercial, or not-for-profit sectors.

### Data availability statement

Data will be made available on request.

### Declaration of interests statement

The authors declare no conflict of interest.

### Additional information

No additional information is available for this paper.

## Appendix A Supplementary data

S1 File Final S1 File Final
