# Supplementary material for: Adaptive profiles of Nellore sheep with reference to farming system and season: physiological, hemato-biochemical, hormonal, oxidative-enzymatic and reproductive standpoint
Source: Heliyon. 2021 May 26;7(5):e07117. doi: 10.1016/j.heliyon.2021.e07117 (PMC8176311; doi:10.1016/j.heliyon.2021.e07117)
Supplement: S1 File Final [file mmc1.pdf]

S1a File. Initial Body weights (kg) of the ewe-lambs

Ewe-lamb No.	Intensive system
1	18
2	17.6
3	17
4	15
5	16.5
6	17
7	16
8	17
9	15
10	16.6
11	17.3
12	14.2
13	17
14	13.5
15	13.8
16	15.5
17	15.8
18	13.3
19	18.2
20	17
**Average**	**16.07**
**SEM**	**0.33**
**Ewe lamb No.**	**Semi-intensive system**
21	15
22	12.5
23	15.5
24	18
25	17.8
26	18
27	16.5
28	17
29	16.5
30	15.6
31	15.6
32	15.4
33	14
34	15.8
35	15
36	16.4
37	15
38	14
39	16
40	13.2
**Average**	**15.64**
**SEM**	**0.33**
**Ewe-lamb No.**	**Extensive system**
41	14.52
42	9.2
43	12.85
44	9.3
45	12.4
46	12.2
47	12
48	14
49	14.4
50	12
51	12.8
52	10.54
53	12
54	13.6
55	11.8
56	12
57	14.5
58	13
59	14
60	11.5
**Average**	**12.43**
**SEM**	**0.35**

S1b File. Initial Body weights (kg) of the rams

Ram No.	Intensive system
1	41.00
2	48.00
3	50.00
4	47.00
5	49.00
6	50.00
**Average**	**47.50**
**SEM**	**1.38**
**Ram No.**	**Semi-intensive system**
7	50.00
8	52.00
9	48.00
10	46.00
11	41.00
12	42.00
**Average**	**46.50**
**SEM**	**1.78**
**Ram No.**	**Extensive system**
13	52.00
14	49.00
15	42.00
16	45.00
17	49.00
18	50.00
**Average**	**47.83**
**SEM**	**1.49**
